# Association between migraine and risk of ocular motor cranial nerve palsy

**DOI:** 10.1038/s41598-022-14621-z

**Published:** 2022-06-22

**Authors:** Soolienah Rhiu, Kyungdo Han, Juhwan Yoo, Kyung-Ah Park, Sei Yeul Oh

**Affiliations:** 1 Department of Ophthalmology, Hallym University School of Medicine, Dongtan Sacred Heart Hospital, Hwaseong, Republic of Korea; 2 Department of Statistics and Actuarial Science, Soongsil University, Seoul, Republic of Korea; 3 Department of Biomedicine and Health Science, The Catholic University of Korea, Seoul, Republic of Korea; 4 Department of Ophthalmology, Samsung Medical Center, Sungkyunkwan University School of Medicine, 81 Irwon-ro, Gangnam-gu, Seoul, 06351 Republic of Korea

**Keywords:** Medical research, Neurology, Risk factors

## Abstract

To assess association between migraines and development of ocular motor cranial nerve palsy (CNP) and finding risk factors using the National Sample Cohort database from the Korea National Health Insurance Service. Data was analyzed from 4,234,341 medical screening examinees aged 20–90 years in 2009. Cox proportional hazard regression analysis was used to the adjusted hazard ratios (HR) for ocular motor CNP according to presence of migraine. Subgroup analysis was performed to evaluate effect of other factors on association of migraine with ocular motor CNP. A total of 5806 participants (0.14% of subjects) developed ocular motor CNP and were assigned to CNP group, 4,048,018 were assigned to control group, with an average of 8.22 ± 0.93 years of follow-up. Incidence of ocular motor CNP increased in migraine group compared to control. After adjusting potential confounding variables, HR for ocular motor CNP was 1.166 (confidence interval [CI] 1.013–1.343) in migraine group. Subgroups of relatively younger age less than 65 years (HR = 1.267, 95% CI 1.067–1.504), male gender (HR = 1.228, 95% CI 1.000–1.122), smokers (HR 1.426, 95% CI 1.127–1.803), and diabetes mellitus patients (HR = 1.378, 95% CI 1.045–1.378) showed a stronger association between migraines and development of ocular motor CNP. Our population-based cohort study demonstrated a significant association between presence of migraines and incidence of ocular motor CNP. Especially, relatively younger age, males, smokers, and diabetes patients with migraines could have a higher risk of developing ocular motor CNP.

## Introduction

The 3rd, 4th, and 6th cranial nerve palsies (CNP), which involve ocular muscles and lead to disabling symptoms including diplopia with or without abnormal head posture, are commonly encountered disease entities in neuro-ophthalmology practice^1–8^. The incidence of these diseases increases with age, and many previous reports revealed that ocular motor CNP had a close association with vasculopathic risk factors such as diabetes mellitus, hyperlipidemia, hypertension, and several diseases of the circulatory system, suggesting microvascular ischemia as the main etiology of ocular motor CNP, especially in elderly patients^9–11^. However, other conditions such as neoplasms, inflammation, infections, giant cell arteritis, trauma, and brainstem infarction were also reported as causes for ocular motor CNP development^1,3–5,12–20^, suggesting that heterogeneous mechanisms might be involved in the development of ocular motor CNP.

Migraine, a very common neurobiological headache disorder, is reported as one of the potential risk factors for ocular motor CNP development in one previous study^21^. In the previous study, a migraine diagnosis was associated with increased risks of developing subsequent, isolated 3rd, 4th, and 6th CNP compared to non-headache matched controls after a mean follow-up of 3.1 years^21^. However there was no mention of which factors increased the risk of ocular motor CNP in migraine patients.

The purpose of this study was to investigate the association between the presence of migraines and ocular motor CNP in the Korean adult population during long-term follow-up period using the National Sample Cohort (NSC) database from the Korea's National Health Insurance Service (NHIS) and identify other factors affecting the association between the risk of ocular motor CNP development and the presence of migraines. Registering in the NHIS has been mandatory for all citizens of Korea since 1989^22^. The NHIS in Korea offers a biennial national health screening program (NHSP) for all beneficiaries aged ≥ 20 years, and especially, all citizens aged 40 years or older are strongly recommended to undergo biennial general health screening^23^. The participation rate in the general health screening program among eligible participants was 74.8% in 2014^24^.

## Materials and methods

### Data sources and study population

In this nationwide, population-based, observational, retrospective cohort study, we utilized medical records from the Korea’s NHIS, a nationwide insurer that covers almost 97% of the entire Korean population. This mandatory, universal program offers comprehensive medical coverage including outpatient and inpatient services, emergency medicine, and prescription drugs for all Korean citizens. The NHIS manages and releases data to the public containing demographic factors, records of medical facility use, and diagnostic codes from the Korean Standard Classification of Diseases (KCD)-7 codes (7th Revision). In the KCD-7, diseases are classified according to the International Classification of Diseases-10 (10th Revision) system. The NHIS also provides a biennial health screening program to people aged ≥ 20 years, which collects past medical history and laboratory test results.

In this study, we analyzed the data from 4,234,341 medical screening examinees aged between 20 to 90 years in 2009. The study participants were followed up until December 31, 2018. After excluding (1) those who have been previously diagnosed with ocular motor CNP (n = 2873), (2) those with missing data (n = 166,941), and (3) those who were newly diagnosed with CNP or died within one year of testing (n = 10,703), we finally picked 4,053,824 eligible individuals. The reason we set the 1-year time lag was to avoid a situation where the causal relationship was reversed.

This study adhered to the tenets of the Declaration of Helsinki and was approved by the Institutional Review Board of Samsung Medical Center (IRB; IRB no. SMC 2020-09-050). The requirement for informed consent from the individual patients was waived from the Institutional Review Board of Samsung Medical Center (IRB; IRB no. SMC 2020-09-050) owing to the retrospective nature of the study and because the data used were public and anonymized under confidentiality guidelines.

### Definition of ocular motor CNP, migraine, and confounders

Ocular motor CNP was identified based on the ICD-10 codes H49.0 (CN3), H49.1 (CN4), or H49.2 (CN6), and codes H06.2 (dysthyroid exophthalmos), E05 (thyrotoxicosis), or G70.0 (myasthenia gravis) were excluded. Migraine was diagnosed based on the ICD-10 codes of G43 for migraine and G431 for migraine with aura. We considered the variables including age, sex, smoking status, drinking amount, physical activity frequency, income status, and body mass index (BMI) as confounders in the relationship between ocular motor CNP and migraines. A standardized self-reported questionnaire was used to gather general health behaviors and lifestyles. The smoking status of the subjects was used to classify them as non-smokers, ex-smokers, or current smokers. The subjects were classified as non-drinkers, mild-to-moderate drinkers, and heavy drinkers according to the amount of alcohol consumed at once. Individuals who consumed more than 30 g of alcohol per day were defined as heavy drinkers. Regular physical activity was defined as doing high-intensity exercise for at least 20 min three times per week or at least 30 min of moderate-intensity exercise five times per week. The low-income level was defined as the bottom 20% of the total population. During the medical examination, height (cm) and weight (kg) were measured using an electronic scale at a medical facility. BMI was calculated as weight (kg) divided by height (m) squared. We also adjusted for the comorbidities of hypertension, diabetes mellitus, and dyslipidemia based on the following criteria. Hypertension was defined as a blood pressure (BP) of ≥ 140/90 mmHg, or a prescription at least once per year for antihypertensive drugs under ICD-10-CM codes I10–I13 or I15. Diabetes mellitus (DM) was defined as a fasting glucose of ≥ 126 mg/dL, or a prescription of one or more antidiabetic drugs at least once per year. Dyslipidemia was defined as a total cholesterol level of 240 mg/dL or higher, or a prescription of a lipid-lowering drug at least once per year under ICD-10-CM code E78.

### Statistical analysis

The baseline characteristics according to the development of ocular motor CNP were compared using the Student’s *t* test for analysis of variance for the continuous variables and the chi-squared test for the categorical variables. All data are presented as mean ± standard deviation (SD). Cox proportional hazard regression analysis was performed for ocular motor CNP development according to the presence of migraine. To adjust for other confounders, we set and compared three models independently. Model 1 was analyzed with no adjustment. Model 2 adjusted for age, sex, smoking status, drinking amount, regularity of physical activity, and income status as potential confounding factors. Model 3 had additional adjustments for the calculated BMI and comorbidities including hypertension, diabetes mellitus, dyslipidemia, and glomerular filtration rate (GFR) in the setting of model 2. The hazard ratio (HR) and confidence interval (CI) were calculated, and we evaluated whether the incidence of ocular motor CNP was increased in the population with migraines compared to the population without them. To evaluate the effect of other factors on the association of migraine with ocular motor CNP, we performed subgroup analysis. We evaluated the effect of age over 65, sex, smoking status, diabetes mellitus, hypertension, and dyslipidemia in the subgroup analysis. The analyses were conducted with SAS version 9.4 (SAS Institute Inc., Cary, NC, USA). A *p*-value of < 0.05 was considered statistically significant.

### Ethics approval

This study adhered to the tenets of the Declaration of Helsinki and was approved by the Institutional Review Board of Samsung Medical Center (IRB; IRB no. SMC 2020-09-050).

### Consent to participate

Consent from the individual patients was waived because the data used were public and anonymized under confidentiality guidelines.

### Consent for publication

Consent for publication was waived because the data used were public and anonymized under confidentiality guidelines.

## Results

### Study population

**Figure 1:**
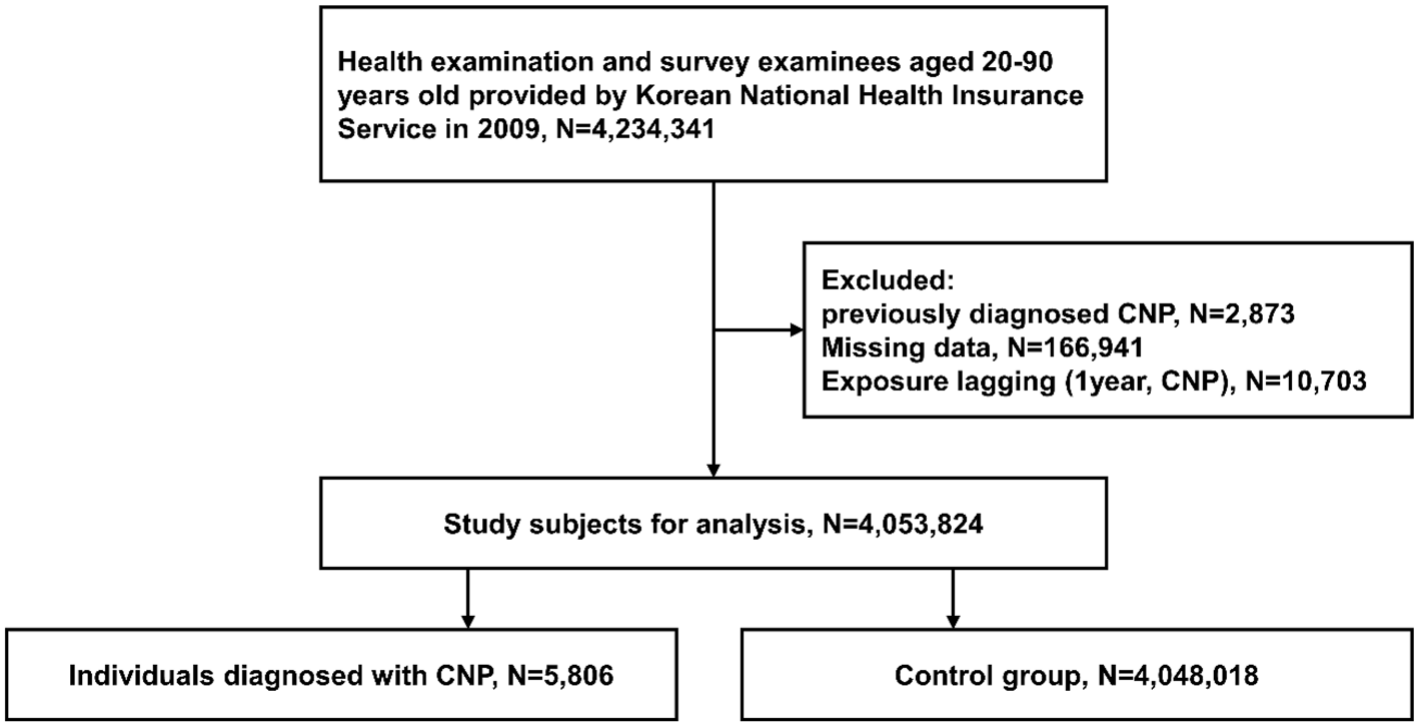
Flowchart of the enrollment process of the current study cohort. *N* numbers, *CNP* ocular motor cranial nerve palsy.

We followed up 4,053,824 eligible subjects for 33,323,032.76 person-years, during which 5806 (0.14% of subjects) developed ocular motor CNP and were assigned to the CNP group and 4,048,018 were assigned to the control group (Fig. 1). The average follow-up duration was 8.22 ± 0.93 years. The baseline characteristics were compared according to the presence of migraine (Table 1). Many factors were significantly associated with the presence of migraine, e.g., age, sex, smoking status, drinking amount, regularity of physical activity, income status, BMI, and comorbidities including hypertension, diabetes mellitus, dyslipidemia, and GFR. We considered these possible confounding variables when evaluating the independent relationship between migraines and ocular motor CNP.

**Table 1:** Baseline characteristics of patients with migraines and controls.

	Controls	Migraine	*p*-value
	N = 3,941,971	N = 111,853	
Age, years	46.91* ± 14.05	51.7* ± 14.24	< 0.0001
Sex, male, N (%)	2,197,348 (55.74)	34,542 (30.88)	< 0.0001
Smoking status, N (%)			< 0.0001
Non-smoker	2,320,248 (58.86)	85,793 (76.7)	
Ex-smoker	573,254 (14.54)	10,899 (9.74)	
Current smoker	1,048,469 (26.6)	15,161 (13.55)	
Drinking amount, N (%)^a^			< 0.0001
Non	2,008,153 (50.94)	76,872 (68.73)	
Mild	1,615,498 (40.98)	30,330 (27.12)	
Heavy	318,320 (8.08)	4651 (4.16)	
Regular exercise, N (%)^b^	717,001 (18.19)	19,195 (17.16)	< 0.0001
Low income, N (%)^c^	686,134 (17.41)	21,096 (18.86)	< 0.0001
Body mass index, kg/cm^d^	23.71* ± 3.22	23.74* ± 3.24	0.0017
Hypertension, N (%)^e^	1,049,701 (26.63)	40,804 (36.48)	< 0.0001
Diabetes mellitus, N (%)^f^	341,977 (8.68)	10,703 (9.57)	< 0.0001
Dyslipidemia, N (%)^g^	708,947 (17.98)	27,972 (25.01)	< 0.0001
GFR, mL/min/1.73 m^2^	87.61* ± 45.1	85.72* ± 36.74	< 0.0001

*N* numbers, *GFR* glomerular filtration rate.

^a^Drinking amount: Individuals who consumed more than 30 g of alcohol per day were defined as heavy drinkers.

^b^Regular exercise: Regular physical activity was defined as doing high-intensity exercise for at least 20 min three times per week or at least 30 min of moderate-intensity exercise five times per week.

^c^Low income: The low-income level was defined as the bottom 20% of the total population.

^d^Body mass index (BMI): BMI was calculated as weight (kg) divided by height (m) squared.

^e^Hypertension (HTN): Hypertension was defined as a prescription at least once per year for antihypertensive drugs under ICD-10-CM codes I10–I13, I15, or blood pressure (BP) of ≥ 140/90 mmHg.

^f^Diabetes mellitus (DM): DM was defined as fasting glucose of ≥ 126 mg/dL or at least one prescription per year for one or more antidiabetic drugs.

^g^Dyslipidemia: Dyslipidemia was defined as a total cholesterol level of 240 mg/dL or higher or at least one prescription per year for a lipid-lowering drug under ICD-10-CM code E78.

*Geometric means.

### The risk of ocular motor CNP according to the presence of migraine

**Figure 2:**
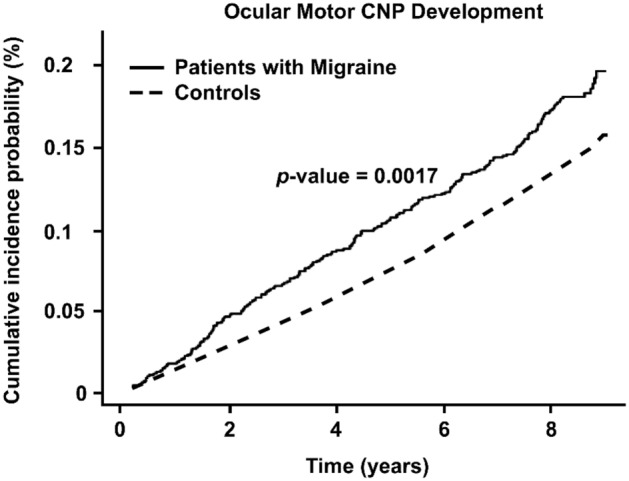
Kaplan–Meier survival curve of outcomes for ocular motor cranial nerve palsy according to the presence of migraine.

The incidence of ocular motor CNP was increased in the population with migraines compared to the population without migraines (Fig. 2). In model 1 without adjustment, the patients with migraines had an HR for ocular motor CNP of 1.252 (95% CI 1.088–1.440) compared to the population without migraines. In model 2, the HR was 1.168 (95% CI 1.015–1.345). After full adjustment for the comorbidities in model 3, the HR was 1.166 (95% CI 1.013–1.343).

### The risk of ocular motor CNP in subgroup analysis according to age, sex, smoking status, and comorbidities including diabetes mellitus, hypertension, and dyslipidemia

Table 2 shows the incidence rates and multivariable-adjusted HRs for the development of ocular motor CNP according to age, sex, smoking status, and comorbidities including diabetes mellitus, hypertension, and dyslipidemia. The analysis was also adjusted for other variables such as age, sex, smoking status, drinking amount, regularity of physical activity, income status, calculated BMI, and comorbidities including hypertension, diabetes mellitus, dyslipidemia, and GFR. The subgroups of relatively younger age less than 65 years (HR = 1.267, 95% CI 1.067–1.504), male gender (HR = 1.228, 95% CI 1.000–1.122), smokers (HR = 1.426, 95% CI 1.127–1.803), and diabetes mellitus patients (HR = 1.378, 95% CI 1.045–1.378) showed a stronger association between migraines and the development of ocular motor CNP compared to the subgroups without them.

**Table 2:** Subgroup analysis for the incidence rate of ocular motor CNP.

Subgroup	Migraine	N	CNP	Incidence rate (/1000)	HR^a^	*p* for interaction
**Age, years**						
Age < 65	No	3,441,551	4034	0.14145	1 (reference)	0.0641
	Yes	88,297	136	0.1819	1.267 (1.067,1.504)	
Age ≥ 65	No	500,420	1569	0.40513	1 (reference)	
	Yes	23,556	67	0.36307	1.019 (0.797,1.302)	
**Sex**						
Male	No	2,197,348	3726	0.20736	1 (reference)	0.401
	Yes	34,542	93	0.32832	1.228 (1,1.51)	
Female	No	1,744,623	1877	0.13015	1 (reference)	
	Yes	77,311	110	0.16951	1.122 (0.926,1.36)	
**Smoking status**^**b**^						
No	No	2,320,248	3023	0.1581	1 (reference)	0.0314
	Yes	85,793	131	0.18256	1.062 (0.891,1.265)	
Yes	No	1,621,723	2580	0.19442	1 (reference)	
	Yes	26,060	72	0.33542	1.426 (1.127,1.803)	
**Diabetes mellitus**^**c**^						
No	No	3,599,994	4281	0.14429	1 (reference)	0.5937
	Yes	101,150	150	0.17716	1.108 (0.94,1.305)	
Yes	No	341,977	1322	0.4859	1 (reference)	
	Yes	10,703	53	0.61982	1.378 (1.045,1.816)	
**Hypertension**^**d**^						
No	No	2,892,270	3163	0.13224	1 (reference)	0.4788
	Yes	71,049	99	0.16554	1.187 (0.971,1.452)	
Yes	No	1,049,701	2440	0.28798	1 (reference)	
	Yes	40,804	104	0.31123	1.150 (0.944,1.4)	
**Dyslipidemia**^**e**^						
No	No	3,233,024	4130	0.15531	1 (reference)	0.0373
	Yes	83,881	148	0.2114	1.268 (1.075,1.495)	
Yes	No	708,947	1473	0.25398	1 (reference)	
	Yes	27,972	55	0.23695	0.963 (0.735,1.262)	

*N* numbers, *CNP* ocular motor cranial nerve palsy, *IR* incidence rate.

^a^The analysis was adjusted for other variables such as age, sex, smoking status, drinking amount, regularity of physical activity, income status, calculated body mass index, and comorbidities including hypertension, diabetes mellitus, dyslipidemia, and glomerular filtration rate.

^b^Smoking status: Smoking status was classified as a non-smoker, ex-smoker, or current smoker.

^c^Diabetes mellitus (DM): DM was defined as fasting glucose of ≥ 126 mg/dL or at least one prescription per year for one or more antidiabetic drugs.

^d^Hypertension (HTN): Hypertension was defined as a prescription at least once per year for antihypertensive drugs under ICD-10-CM codes I10–I13, I15, or a BP of ≥ 140/90 mmHg.

^e^Dyslipidemia: Dyslipidemia was defined as a total cholesterol level of 240 mg/dL or higher or at least one prescription per year for a lipid-lowering drug under ICD-10-CM code E78.

## Discussion

This is a nationwide, population-based cohort study, demonstrating risk factors of ocular motor CNP in migraine patients. Migraine patients with young age, male gender, current smoking status, and diabetes mellitus showed a higher risk of ocular motor CNP development compared to those without them. The incidence of ocular motor CNP among Koreans appeared to be positively associated with the presence of migraine, which agrees with a previous study in Taiwan^21^. In our study, these outcomes were significant with or without adjustment for various confounders including age, sex, smoking status, drinking amount, regularity of physical activity, BMI, income status, and the comorbidities of hypertension, diabetes mellitus, dyslipidemia, and GFR.

Migraine is a very common neurobiological headache disorder that is caused by increased excitability of the central nervous system (CNS) and is characterized by various combinations of neurological, gastrointestinal, and autonomic changes^25^. It ranks high among global disabilities^26^. As the association between migraines and ocular motor CNP was raised in only one previous study^21^, there have not been extensive discussions on this association. The authors in the previous study suggested that the development of ocular motor CNP in patients with migraines may have been due to microvascular ischemia^21^. Although the exact mechanism of migraine generation is not completely understood, the now generally accepted mechanism for migraine headaches is the neurovascular hypothesis^26–33^. Study results have supported that migraine with aura begins with an event evolving cortical spreading depression (CSD), which in turn, activates the trigeminovascular system, the system that allows for nociceptive signals from the meningeal blood vessels to transmit to higher centers of the CNS^27–30^. When activated, the trigeminal sensory nerves trigger the release of vasoactive neuropeptides, which results in vasodilation and dural plasma extravasation, leading to neurogenic inflammation^26^. Pain impulses are then transmitted through the trigeminovascular system to the trigeminal nucleus caudalis, then to higher cortical pain centers in the brain^26,34,35^. CSD may predispose to CNS dysfunction by activating an inflammatory cascade, by hypoperfusion as spreading oligemia, and by a neurovascular coupling failure to provide a sufficient blood flow increase for the energy demand increase in CSD^31,36–40^. We could postulate that these complex cascades including neurogenic inflammation and the failure of neurovascular coupling may contribute to the development of ocular motor CNPs, although further studies are needed to validate this theory. Also, regarding the previous evidence suggesting that the vascular system in migraine patients is impaired at a systemic level including impaired compliance of the arterial system and endothelial dysfunction such as altered flow-mediated dilation and reduced endothelial progenitor cells^41^, we could not rule out the possibility that a peculiar vascular vulnerability of migraine patients contributes both to the development of migraines and the development of CNP.

In our study, we found that especially, migraine patients with young age, male gender, current smoking status, and diabetes mellitus showed a higher risk of ocular motor CNP development compared to those without them, which has not been reported in previous studies. Gender differences in migraine and comorbidities have been mainly studied for ischemic stroke^42–44^. The previous studies, including five meta-analyses, reported a significant association between migraines, particularly migraines with aura, and the development of stroke^42,44–47^, and those associations were reported to be stronger in women, and women younger than 45 years^42–44^. The authors of the previous studies on the association between ischemic stroke and migraines reported that because the prevalence of migraines is three times lower in men than in women, the association was more uncertain for men^42^. However, no previous data are available on gender differences in the risk of the ocular motor CNP in migraine patients. Our study showed a male gender in patients with migraines increased the risk of CNP development. Although it needs to be confirmed through other studies, our findings suggested the possibility that male gender, along with young age and diabetes mellitus can be potential risk factors for ocular motor CNP development in migraine patients.

There were several limitations to this study. First, due to the retrospective study design, we could not confirm a causal relationship between migraines and ocular motor CNP. Although we took into consideration confounders in this study, all confounders such as current medications or all past medical history could not be removed due to the nature of the NHIS. Second, we only included a Korean adult population. Therefore, the application of these data to other races may need to be qualified.

In conclusion, in this relatively long-term population-based cohort study, we demonstrated a significant association between the presence of migraines and the incidence of ocular motor CNP. Relatively younger age, male gender, smoking, and diabetes patients with migraines could have a higher risk of developing ocular motor CNP. The possible pathophysiology underlying these associations, such as neurogenic inflammation related to migraine, needs to be explored through further research.

## Data Availability

The datasets generated during and/or analyzed during the current study are available from the corresponding author on reasonable request.
